# The effects of PEGylation on LNP based mRNA delivery to the eye

**DOI:** 10.1371/journal.pone.0241006

**Published:** 2020-10-29

**Authors:** Renee C. Ryals, Siddharth Patel, Chris Acosta, Madison McKinney, Mark E. Pennesi, Gaurav Sahay

**Affiliations:** 1 Department of Ophthalmology, Casey Eye Institute, Oregon Health & Science University, Portland, Oregon, United States of America; 2 Department of Pharmaceutical Sciences, College of Pharmacy, Oregon State University, Portland, Oregon, United States of America; University of Florida, UNITED STATES

## Abstract

Gene therapy is now an effective approach to treat many forms of retinal degeneration. Delivery agents that are cell-specific, allow for multiple dosing regimens, and have low immunogenicity are needed to expand the utility of gene therapy for the retina. We generated eight novel lipid nanoparticles (LNPs) ranging in size from 50 nm to 150 nm by changing the PEG content from 5% to 0.5%, respectively. Subretinal injections of LNP-mRNA encoding luciferase revealed that 0.5% PEG content within nanoparticles elicits the highest expression. Similar injections of LNP delivered cre mRNA into Ai9 mice revealed cell-specific protein expression in the retinal pigment epithelium (RPE), confirmed by fundus photography and immunohistochemistry of whole globe cross-sections. To investigate mechanisms of LNP delivery to the eye, we injected mCherry mRNA using the subretinal approach in *apoE*^*-/-*^ and *Mertk*^*-/-*^ mice. RPE transfection was observed in both mouse models suggesting that LNP intracellular delivery is not solely dependent on apolipoprotein adsorption or phagocytosis. To investigate LNP penetration, particles were delivered to the vitreous chamber via an intravitreal injection. The 0.5% PEG particles mediated the highest luciferase activity and expression was observed in the Müller glia, the optic nerve head and the trabecular meshwork, but failed to reach the RPE. Overall, particles containing less PEG (~150 nm in size) mediated the highest expression in the eye. Thus far, these particles successfully transfect RPE, Müller cells, the optic nerve head and the trabecular meshwork based on route of administration which can expand the utility of LNP-mediated gene therapies for the eye.

## Introduction

Despite major advancements in restoring vision, there are still an estimated 285 million vision-impaired and 39 million blind people around the world [1]. In part, these large numbers exist because there are still no treatments for some of the most devastating blinding diseases including advanced glaucoma, atrophic macular degeneration, advanced diabetic retinopathy, myopia, and inherited retinal degeneration [1]. These disorders have become the focus of ocular gene therapy and effective treatments are being developed. For example, in 2017, the FDA approved an adeno-associated virus (AAV2)-based gene augmentation therapy, voretigene neparvovec (Luxturna^tm^), for inherited retinal dystrophy patients harboring biallelic mutations in RPE65 [2, 3]. This is now one of the six diseases treated with a cell or gene therapy in the USA, highlighting the safety and efficacy of subretinal AAV delivery for ocular gene therapy.

Even with the recent surge in pre-clinical and clinical investigations evaluating gene therapies for the eye, many advances are still required [4, 5]. Specific to treating the retina, clinicians and scientists are in much debate over the visual impact of detaching the fovea and/or a fragile degenerating retina during a subretinal injection [6]. The intravitreal injection, which does not damage or detach the retina, offers a safer ocular route for gene delivery [7]. However, when shifting to the intravitreal delivery route, high viral titers will be required increasing immune activation and potential adverse events [5]. Lipid based nanoparticles (LNP), being the most clinically advanced, are an attractive alternative gene delivery vehicle due to their modularity and safety profile [8, 9]. In 2018, the FDA approved the first ever LNP based formulation of siRNA, Onpattro^®^, for the treatment of hereditary transthyretin amyloidosis [10, 11]. Subsequently, in 2020, a LNP-encapsulated mRNA-based vaccine was developed and evaluated in clinical trials for SARS-CoV-2 [12]. LNPs are comprised of structural lipids, cationic or ionizable lipids, PEG-lipids and cholesterol (50:10:1.5:38.5% mol ratios), all which can be rationally designed for optimal cell-specific gene delivery. Previously, we evaluated 11 different cationic or ionizable lipids for their ability to transfect the retina post-subretinal delivery [13]. We found that LNPs containing the ionizable lipid, MC3 (pKa = 6.5), most efficiently transfect the retinal pigment epithelium (RPE).

When transitioning to an intravitreal delivery method, there are barriers to the neural retina and the RPE that must be considered. The vehicle, once delivered to the vitreous chamber, will have to move through the vitreous humor, which is composed of collagen, hyaluronan, glycosaminoglycans, and a cocktail of various proteins which restrict diffusion [14]. If intact, the vehicle must then penetrate an inner limiting membrane (ILM), which is a complex network of collagen, fibronectin, laminin and proteoglycans restricting permeability of substances into the retina [15]. Finally, to reach the RPE, the vehicle must penetrate the outer limiting membrane (OLM), which restricts transport and permeability with both tight and adherens junctions [16]. Many intravitreal nanoparticles have been developed with the intent to overcome these barriers and treat ocular conditions including inflammation, glaucoma, angiogenesis and retinal degeneration [17]. Specific to nucleic acid delivery to the retina, Apaolaza et al. developed solid-lipid nanoparticles that successfully delivered condense plasmid DNA encoding human retinaoschisin (RS1) to multiple layers in the retina that improved the retinal structure in a mouse model of x-linked retinoschisis [18]. In addition, Puras et al. constructed DNA-loaded lipoplexes that demonstrated successful transfection of eGFP in the inner retina post-intravitreal delivery in the Sprague-Dawley rats [19]. To enhance antisense oligonucleotide permeability and retinal distribution, Tai et al. used penetratin-modified hydroxyl-terminated polyamidoamine, which resulted in RPE uptake up to 8 hours post-intravitreal delivery [20]. Despite these advances, we still lack nanoparticles that can efficiently deliver nucleic acids for gene augmentation, editing and silencing to the photoreceptors and RPE post-intravitreal delivery. To further the development of intravitreal nanoparticles, our strategy is 1) to utilize and improve upon the most clinically advanced LNP system that has already been FDA approved, enhancing translatability and 2) to deliver mRNA instead of DNA which enhances transfection efficiency by removing the need to shuttle DNA to the nucleus and undergo transcription. Our LNPs will be optimal for gene editing approaches requiring transient gene expression. However, studies that elucidate the mechanisms responsible for LNP penetration and internalization in the retina post-intravitreal injection are needed to rationally design enhanced nanotherapeutics.

Upon characterizing many of the intravitreal nanoparticles developed thus far and assessing their effectiveness, four main parameters continuously affect their intraocular distribution and elimination: size, charge, stability and ligands [17]. It has been demonstrated that size affects the half-life of intravitreally injected nanoparticles. Specifically, for polystyrene nanoparticles smaller particles sizes ~ 50 nm had the highest half-life [21]. Furthermore, size impacts the process for internalization. In general, particles <500 nm are internalized via endocytosis, whereas particles >1000 nm utilize phagocytosis [22, 23]. Stability and size are often linked as the particle size varies upon disassembly or aggregation of the particles. If nanoparticles are unstable, they will fall apart upon entry, and the dissociated components can be rapidly eliminated [24]. Thus, particles need to be stable enough to travel to the cell of interest, but then have the ability to disassemble once internalized in the cell of interest. It has been shown that the hydrophilic polymer, polyethylene glycol (PEG), which is a commonly used excipient to increase *in vivo* stability, evade recognition by the reticuloendothelial system, prolong circulation half-life and reduce immunogenicity, can be easily adjusted to impact particle size, stability and intracellular delivery [25]. To evaluate the important parameters of size and stability for intravitreal delivery, we generated 8 novel LNPs that varied in size by changing mol % of PEG. We found that larger particles (~150 nm), containing less PEG-lipid (0.5%), mediated the highest levels of gene expression post-subretinal and intravitreal delivery. Most intriguingly, these particles successfully transfect RPE, Müller glia, the optic nerve head and the trabecular meshwork based on route of administration which can expand the utility of LNP-mediated gene therapies for the eye.

## Materials and methods

### Materials

Dlin-MC3-DMA (MC3) was purchased through BioFine International Inc. 1,2-distearoyl-sn-glycero-3-phosphocholine (DSPC) was acquired from Avanti Polar Lipids. Cholesterol and 1,2-dimyristoyl-rac-glycero-3-methoxypolyethylene glycol-2000 (DMG-PEG2k) was purchased from MP Biomedicals and NOF America Corporation, respectively. Pierce D-Luciferin was purchased from Thermo Fisher Scientific. Firefly luciferase (L-7202), Cre (L-7211) and mCherry (L-7203) mRNA were all purchased from Trilink Biotechnologies.

### Animals

Albino BALB/c (Cat# 000651), Ai9 (Cat# 007909), *apoE*^*-/-*^ (Cat# 002052), *Mertk*^*-/-*^ (Cat# 011122) and C57BL6 (Cat# 000664) mice were either bred in house or purchased from The Jackson Laboratory (Bar Harbor, ME, USA). Ai9 is a Cre reporter tool designed to have a *lox*P-flanked STOP cassette preventing transcription of tdTomato under the control of a ubiquitous promoter. Following Cre-mediated recombination Ai9 mice express robust tdTomato [26]. Both male and female mice aged 1 to 6 months were used in experiments. All the experimental procedures followed the protocols approved by the Institutional Animal Care and Use Committee at Oregon Health & Science University and were in adherence to the Association for Research in Vision and Ophthalmology (ARVO) Statement for the Use of Animals in Ophthalmic and Vision Research.

### Nanoparticle formulation and characterization

LNPs were formulated via microfluidic mixing of one-part ethanol phase (containing the lipids) and three parts aqueous phase (containing the mRNA). The ethanol phase contained the MC3, DSPC, DMG-PEG2k, and cholesterol at a molar ratio of 50:10:1.5:38.5, respectively. For synthesizing novel particles, the percentage of DMG-PEG2k was changed from 1.5% to 0.5%, 3% or 5% while cholesterol was changed from 38.5% to 39.5%, 37.0% or 35%, respectively. For the second set of particles, DMG-PEG2k varied from 0.5% to 5% while DSPC was changed from 10% to 11%, 8.5% or 6.5%, respectively (Table 1). The aqueous phase consisted of the mRNA in 50 mM Citrate buffer pH 4. Following microfluidic mixing, the nanoparticle solution was subjected to buffer exchange with PBS (pH 7.2) and concentrated using Amicon Ultra-4100k MWCO (EMD Millipore) centrifugal filters. The nanoparticles were stored at 4°C until diluted for injections. The LNPs were characterized for hydrodynamic radius (nm) and polydispersity index (PDI) using dynamic light scattering (Zetasizer Nano ZSP, Malvern Instruments), while mRNA encapsulation efficiency was measured using a modified Quant-iT RiboGreen RNA reagent (Life Technologies).

**Table 1:** LNP Components and characteristics.

LNP #	DMG-PEG (%)	Cholesterol (%)	DSPC (%)	MC3 (%)	Encapsulation Efficiency (%)
1	0.5	39.5	10	50	97.8
2	1.5	38.5	10	50	96.6
3	3	37	10	50	98.4
4	5	35	10	50	94.8
5	0.5	38.5	11	50	96.2
6	1.5	38.5	10	50	95.8
7	3	38.5	8.5	50	97.2
8	5	38.5	6.5	50	97.0

LNP-Lipid nanoparticle, DMG-PEG-1,2-dimyristoyl-rac-glycero-3-methoxypolyethylene glycol-2000, DSPC-1,2-distearoyl-sn-glycero-3-phosphocholine, MC3-Dlin-MC3-DMA.

### Injections

Prior to injections, mice were topically administered 0.5% proparacaine, 1% tropicamide, and 2.5% phenylephrine and anesthetized with ketamine (100 mg/kg)/xylazine (10 mg/kg). Our subretinal injection procedure has been previously described elsewhere [13]. For intravitreal injections, 2.5% hypromellose was placed over the eye and a 30-gauge needle was used to make an incision in the limbus. Going through the scleral incision in the limbus, using a hamilton syringe with a 33-gauge 20° beveled needle, 1.5 μl of PBS, LNP-Luciferase, LNP-Cre or LNP-mCherry was delivered to the vitreous chamber. A 2% fluorescein solution was added to the PBS or LNPs so injection quality could be observed and documented. For subretinal injections, 200 ng (1 μl, 200 ng/μl) of mRNA was delivered. For intravitreal injections, a low dose of 300 ng (1.5 μl, 200 ng/μl) of mRNA was evaluated first. To maximize mRNA delivery, particles were concentrated to ~1000 ng/μl and a high dose of 1.5 μg (1.5 μl, 1000 ng/μl) of mRNA was delivered.

### *In-vivo* bioluminescent imaging

Mice were injected intraperitoneally with 150 mg of Luciferin/kg body weight according to manufacturer’s protocol [13]. Bioluminescent imaging was conducted on the IVIS Spectrum In Vivo Imaging System (PerkinElmer). Image analysis for region of interest (ROI) measurement was performed on Living Image Software (PerkinElmer) and was reported as average radiance (the sum of the radiance from each pixel inside the ROI/number of pixels or super pixels (photons/sec/cm^2^/sr)). Sample sizes were 4 to 6 per group.

### Fundus photography

Live, *in-vivo* retinal imaging was performed with the Micron IV (Phoenix Research Laboratories, Pleasanton, CA). To observe general retinal health, bright field images were acquired. To capture tdTomato, a 534/42 nm BrightLine^Ⓡ^ single-band bandpass filter (Semrock, Rochester, NY) was used. Light intensity, exposure and gain were kept consistent across all RFP images. To quantify tdTomato expression, pixel intensity was measured for each image over a consistent area (640,000 pixels^2^) using ImageJ (version 1.49; National Institutes of Health, Bethesda, MD). There were 3 to 6 images per group.

### Immunohistochemistry (IHC)

At specified time points, mouse eyes were enucleated and fixed in 4% paraformaldehyde (pH 7.4) overnight at 4°C. After fixation, oriented eyes were placed in cassettes, processed and embedded for sectioning (Tissue-Tek VIP^®^6, Tissue-Tek^®^ TEC™ 5, Sakura Finetek USA, Inc., CA). Whole globe cross-sections were cut with a microtome to a thickness of 4 microns. To initiate staining, sections were dipped in 100% Xylene, 100% ethanol, 95% ethanol, 80% ethanol, running water and deionized water to deparaffinize the tissue. All sections were then washed with phosphate buffered saline (PBS), permeabilized with 0.3% triton for 10 minutes and blocked with 1% BSA for 1 hour. A primary antibody consisting of an anti-RFP antibody (1:500, rabbit, ab62341) in 1% BSA covered the sections overnight at 4°C. The next day, sections were washed with PBS and incubated in secondary antibody (donkey anti-rabbit Alexa 594, 1:800, Cat#A21207, Life technologies, Eugene, OR). Confocal images were obtained with the TCS SP8 X (Leica Microsystems, Buffalo Grove, IL). Z-stacks (spanned 10 μm with 1 μm interval) were collected using a 40X objective, and maximum intensity projections were reported. Sample size was 3 to 5 per group.

### Statistical analysis

For *in-vivo* bioluminescent imaging measurements from BALB/c mice, differences between groups at each time point were calculated using a two-way ANOVA, Tukey’s multiple comparisons test (Prism 8 software, GraphPad Software, La Jolla, CA). An unpaired t-test was used for *in-vivo* bioluminescent imaging analysis comparing C57BL6 and *apoE*^*-/-*^ mice. For tdTomato quantification from fundus images, intensity values were transformed and reported as fold change to PBS-injected animals. An ordinary one-way ANOVA, with Tukey’s correction for multiple comparisons test was used for comparisons between groups. Data are presented as mean ± SD. A P < 0.05 was considered as statistically significant.

## Results

**Fig 1:**
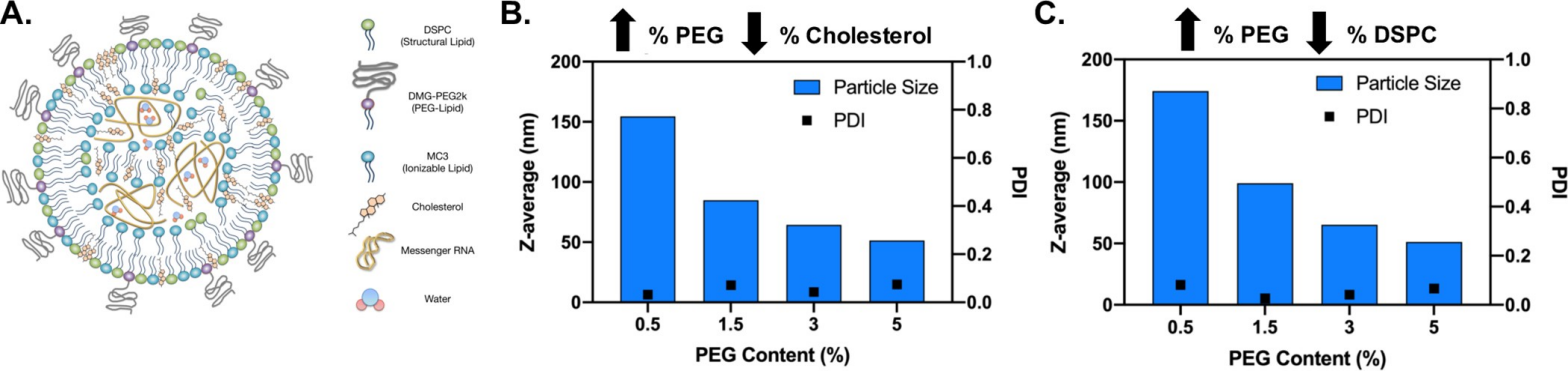
LNP characterization. (A) Schematic of an LNP listing all major components. (B) Bar graph showing the size and PDI of one set of particles when percentage of PEG is increased and modulated against cholesterol. (C) Bar graph showing size and PDI of one set of particles when percentage of PEG is increased and modulated against DSPC. DSPC-1,2-distearoyl-sn-glycero-3-phosphocholine, DMG-PEG2k-1,2-dimyristoyl-rac-glycero-3-methoxypolyethylene glycol-2000, MC3-Dlin-MC3-DMA, PDI-polydispersity index.

LNPs were generated with DSPC (structural lipid), MC3 (ionizable lipid), cholesterol and DMG-PEG2k (PEG-lipid) (Fig 1A). In order to vary LNP size, mol percentages of PEG-lipid were increased from 0.5% - 5% [25]. The increase in mol percentage of PEG was off-set by a decrease in cholesterol (referred to as modulated against cholesterol) or phospholipid (referred to as modulated against DSPC) in order to control for the impact of other components on delivery. PEG-lipid variations from 0.5% to 5% generated particles sized 150 nm to 50 nm, respectively (Fig 1B and 1C). Regardless of the modifications, particle formulations were successful as PDI values were <0.1 and encapsulation efficiency averaged 97% for the eight particles evaluated (Fig 1 and Table 1).

**Fig 2:**
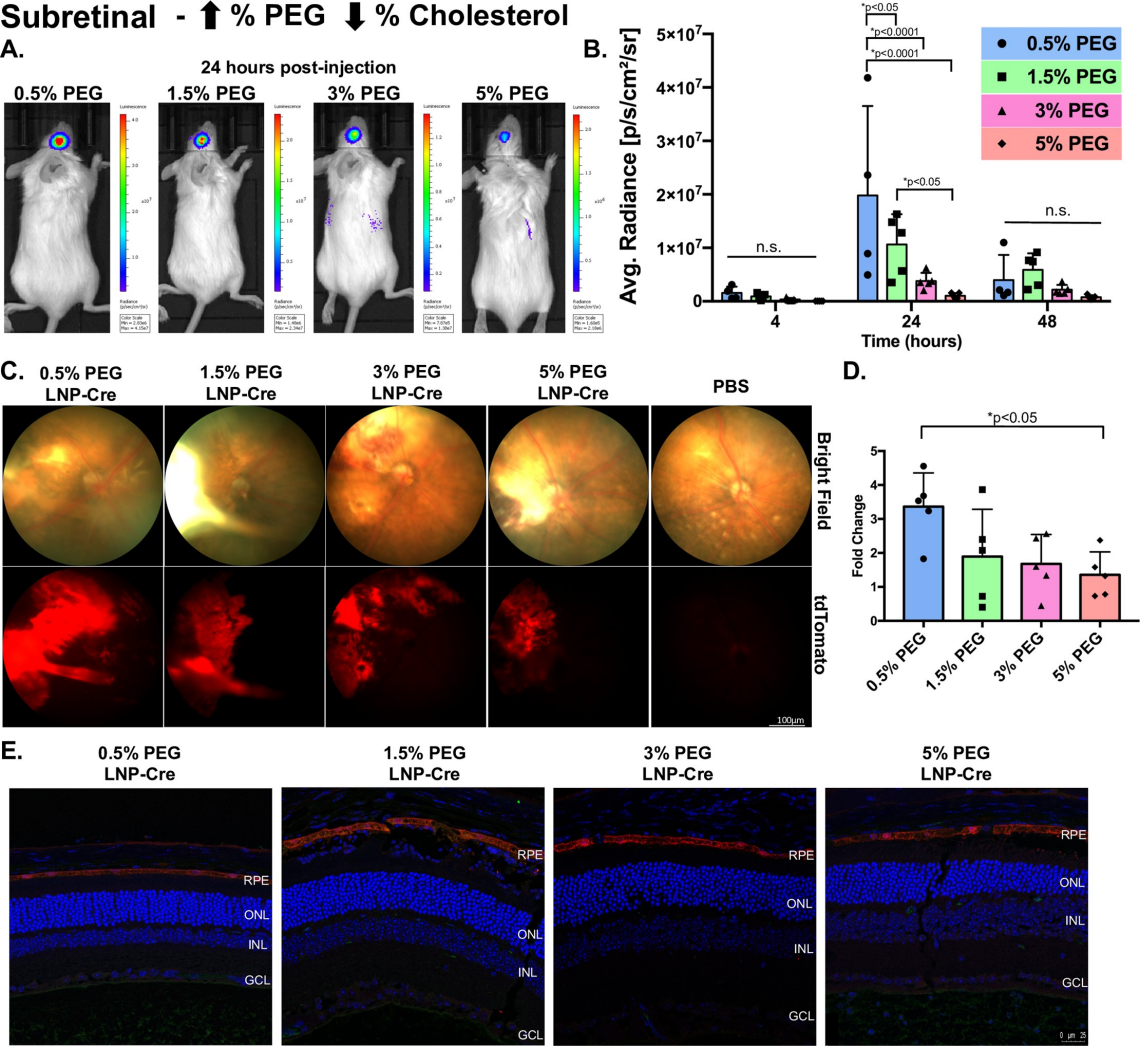
The effects of LNP size and cholesterol modifications post-subretinal injection. (A) Representative images demonstrating luciferase activity in the eye 24 hours post-injection. (B) Luciferase activity plotted for each group over time. Data are presented as mean ± SD. A two-way ANOVA, Tukey’s multiple comparisons test was used for analysis. *p<0.05. n = 3–6. (C) Representative bright field (top) and tdTomato (bottom) fundus images for each group taken 7 days post-injection. (D) Quantification of tdTomato intensity, represented as a fold change compared to PBS. Data are presented as mean ± SD. An ordinary one-way ANOVA, with Tukey’s correction for multiple comparisons test was used for comparisons. *p<0.05. n = 3–6. (E) Representative confocal images of immunohistochemistry showing RFP expression in the RPE for all groups. n.s.—not significant, LNP- lipid nanoparticle, RFP-red fluorescent protein, RPE-retinal pigment epithelium, ONL- outer nuclear layer, INL-inner nuclear layer, GCL- ganglion cell layer.

**Fig 3:**
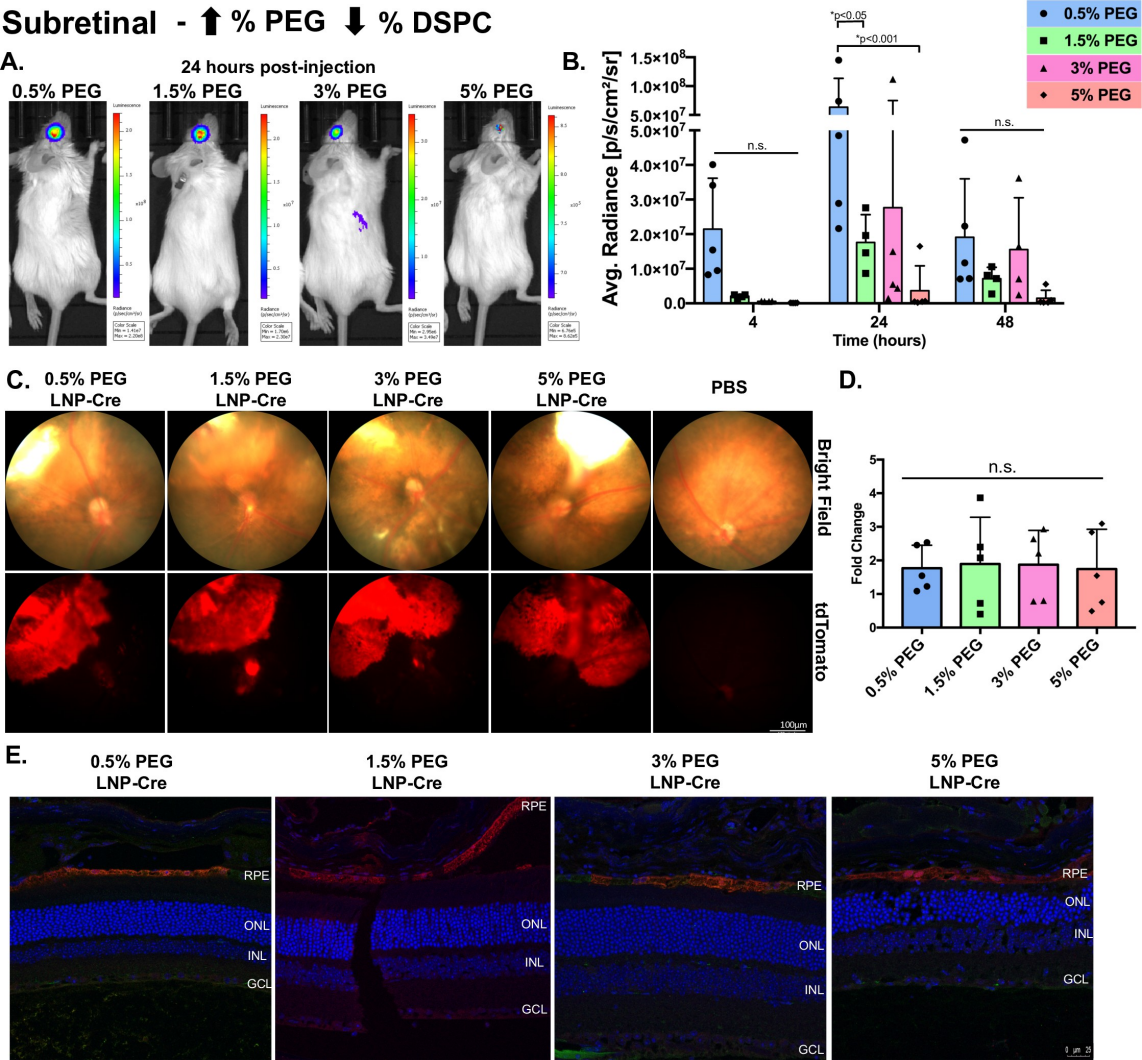
The effects of LNP size and DSPC modifications post-subretinal injection. (A) Representative images demonstrating luciferase activity in the eye 24 hours post-injection. (B) Luciferase activity plotted for each group over time. Data are presented as mean ± SD. A two-way ANOVA, Tukey’s multiple comparisons test was used for analysis. *p<0.05. n = 3–6. (C) Representative bright field (top) and tdTomato (bottom) fundus images for each group taken 7 days post-injection. (D) Quantification of tdTomato intensity, represented as a fold change compared to PBS. Data are presented as mean ± SD. An ordinary one-way ANOVA, with Tukey’s correction for multiple comparisons test was used for comparisons. n = 3–6. (E) Representative confocal images of immunohistochemistry showing RFP expression in the RPE for all groups. n.s.—not significant, LNP- lipid nanoparticle, RFP-red fluorescent protein, RPE-retinal pigment epithelium, ONL- outer nuclear layer, INL-inner nuclear layer, GCL- ganglion cell layer.

Since the subretinal injection places the LNPs in close proximity to the RPE, we utilized this method to understand how PEGylation and size may impact intracellular delivery (Figs 2 and 3). First, LNP kinetics were measured with a luciferase assay (Figs 2A and 3A, S1 Fig). In general, for all particles, luciferase activity in the eye was measurable at 4 hours post-injection, increased to a maximum level at 24 hours post-injection and decreased by 48 hours post-injection (Figs 2B and 3B). At 24 hours post-injection, 0.5% PEG particles with corresponding cholesterol modifications demonstrated significant 1.9-fold, 5.2-fold and 18.9-fold increases in luciferase activity compared to 1.5%, 3% and 5% PEG particles, respectively (Fig 2B, p<0.05). At the same time point, 0.5% PEG particles with corresponding DSPC changes mediated significantly higher levels of luciferase activity compared to 1.5% and 5% PEG particles (3.6-fold and 17.3-fold, respectively) (Fig 3B, p<0.05). Although 0.5% PEG particles showed at 2.3-fold increase in luciferase activity compared to 3% PEG particles, this difference was not significant due to the variation in activity meditated by the 3% PEG particles (Fig 3B). When comparing the luciferase expression between 0.5% PEG particles modulated against cholesterol with 0.5% PEG particles modulated against DSPC, modifications in DSPC mediated a 6-fold, 1.9-fold and 3.9-fold increase in expression compared to cholesterol at 4-, 24- and 48-hours post-injection, respectively. To determine the location of protein expression in the eye, cre mRNA was subretinally injected in Ai9 mice, which results in tdTomato fluorescence upon successful transfection and Cre-mediated recombination [26]. For all eight particles, fundus images showed a cobble stone pattern of expression, traditional of RPE morphology (Figs 2C and 3C). When tdTomato intensity was analyzed, 0.5% PEG particles modulated against cholesterol had 3.4-fold increased tdTomato intensity compared to PBS control and 2-fold increased tdTomato intensity compared to 5% PEG, which was statistically significant (Fig 2D, p<0.05). In contrast, regardless of the PEG content, particles modulated against DSPC averaged a 1.8-fold increase in tdTomato intensity compared to PBS controls, but no statistical differences between the groups were identified (Fig 3D). Immunohistochemistry, performed with a red fluorescent protein (RFP) antibody, demonstrated that protein expression was localized to the RPE for all particles, regardless of modifications (Figs 2E and 3E).

**Fig 4:**
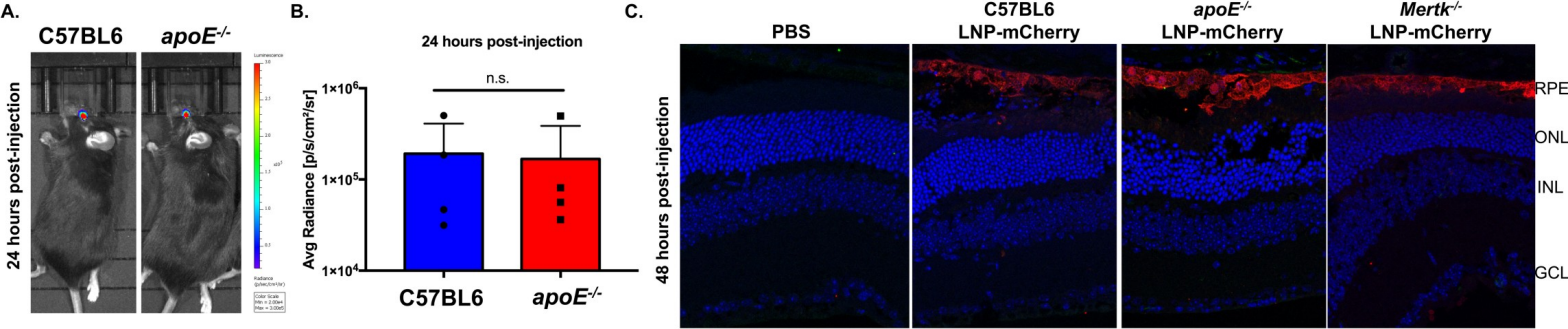
LNP-mediated RPE expression is not dependent on apoE or the MERTK receptor. (A) Representative images demonstrating luciferase activity in C57BL6 and *apoE*^*-/-*^ eyes 24 hours post-injection. (B) Luciferase activity plotted for both groups at 24 hours post-injection. Data are presented as mean ± SD. An unpaired t-test was used for analysis. n = 4. (C) Representative confocal images of immunohistochemistry showing mCherry expression in the RPE of C57BL6, *apoE*^*-/-*^ and *Mertk*^*-/-*^ mice. n.s.—not significant, PBS-phosphate buffered saline, LNP- lipid nanoparticle, RPE-retinal pigment epithelium, ONL- outer nuclear layer, INL-inner nuclear layer, GCL- ganglion cell layer.

After systemic delivery of LNPs, apolipoprotein E (apoE) binds LNPs in the blood, which facilitates apoE-mediated uptake by low-density lipoprotein (LDL) receptors (LDLR) into hepatocytes. This was established by observing diminished LNP transfection efficiency in both *apoE*^*-/-*^ and *LDLR*^*-/-*^ mice [27]. To determine if this mechanism plays a role in LNP uptake in the RPE, the FDA approved LNPs (MC3, DSPC, DMG-PEG2k, and cholesterol at a molar ratio of 50:10:1.5:38.5) containing luciferase mRNA were subretinally injected into *apoE*^*-/-*^ mice (Fig 4A and 4B). Luciferase expression was evident in *apoE*^*-/-*^ mouse eyes at 24 hours post-injection and was not significantly different from expression measured in C57BL6 control animals (Fig 4A and 4B, p>0.05). These data suggested that apoE is not functioning as an endogenous targeting ligand for LNP uptake in the RPE. Once a day, RPE cells phagocytose and digest the shedding rod and cone photoreceptor outer segment tips making RPE cells the most actively phagocytic cells in the body [28]. We hypothesized that if phagocytosis was blocked, LNP entry in the RPE would diminish. LNPs containing mCherry mRNA were subretinally injected in *Mertk*^*-/-*^ mice, in which the main receptor responsible for phagocytosis is dysfunctional and leads to the accumulation of shed photoreceptor outer segments in the subretinal space [29]. Immunohistochemistry, probing for mCherry, demonstrated robust protein expression localized to the RPE in *Mertk*^*-/-*^ and *apoE*^*-/-*^ mice (Fig 4C) verifying that neither phagocytosis nor apoE adsorption are solely responsible for LNP intracellular delivery in the RPE.

**Fig 5:**
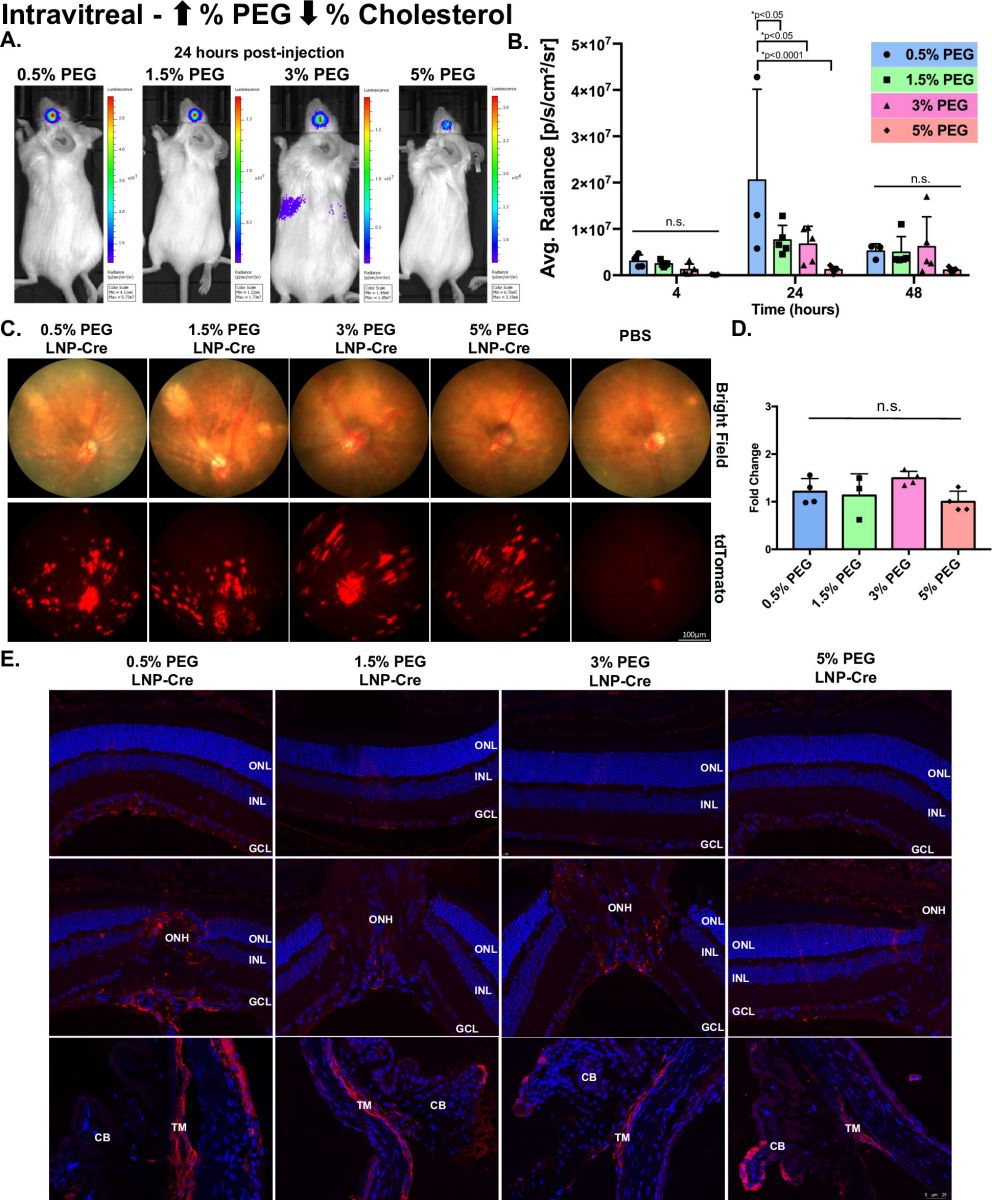
The effects of LNP size and cholesterol modifications post-intravitreal injection. (A) Representative images demonstrating luciferase activity in the eye 24 hours post-injection. (B) Luciferase activity plotted for each group over time. Data are presented as mean ± SD. A two-way ANOVA, Tukey’s multiple comparisons test was used for analysis. *p<0.05. n = 3–6. (C) Representative bright field (top) and tdTomato (bottom) fundus images for each group taken 7 days post-injection. (D) Quantification of tdTomato intensity, represented as a fold change compared to PBS. Data are presented as mean ± SD. An ordinary one-way ANOVA, with Tukey’s correction for multiple comparisons test was used for comparisons. n = 3–6. (E) Representative confocal images of immunohistochemistry showing RFP expression in the Müller glia, the ONH and the TM for all groups. n.s.—not significant, LNP- lipid nanoparticle, RFP-red fluorescent protein, ONL- outer nuclear layer, INL-inner nuclear layer, GCL- ganglion cell layer, ONH-optic nerve head, TM-trabecular meshwork, CB-ciliary body.

**Fig 6:**
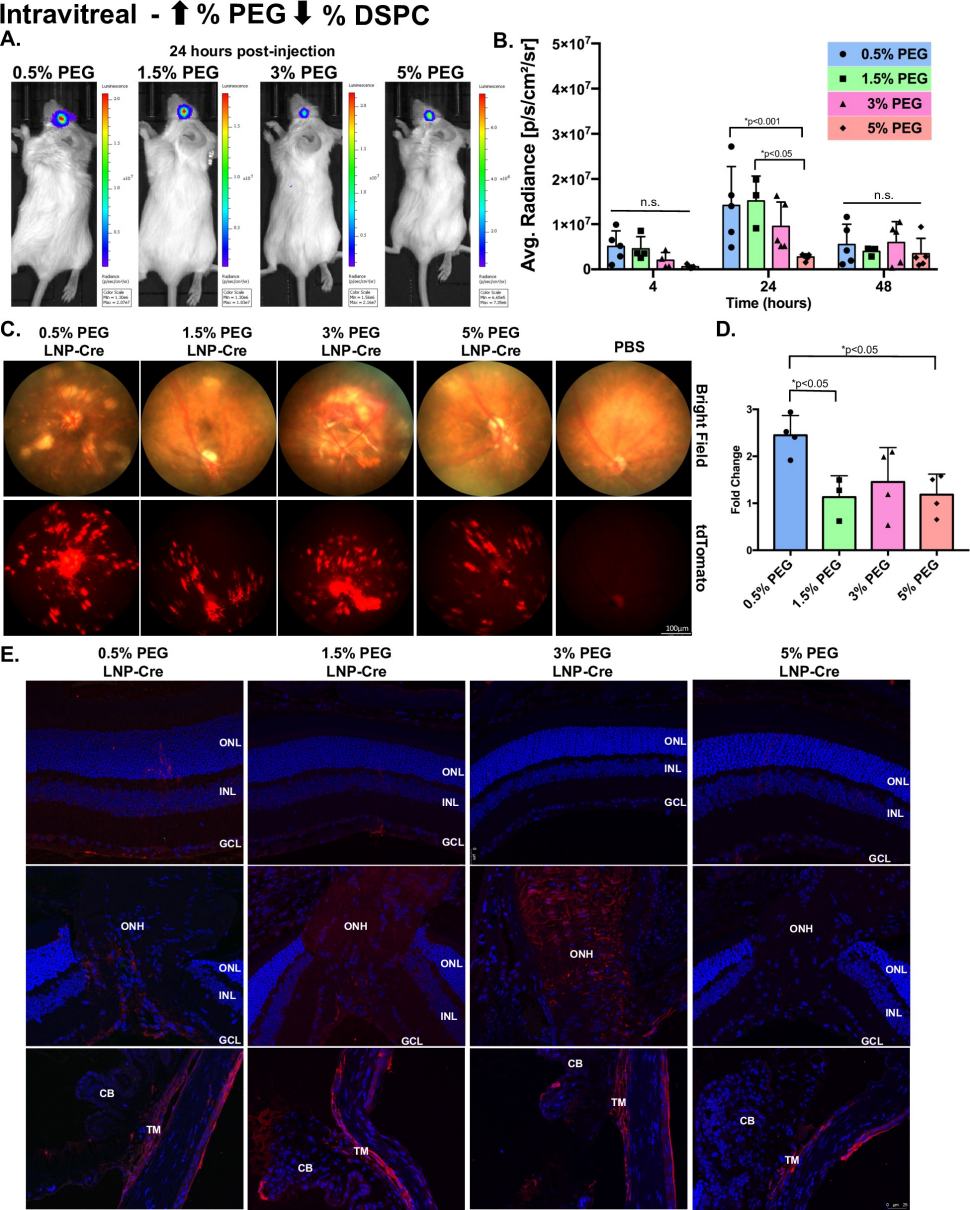
The effects of LNP size and DSPC modifications post-intravitreal injection. (A) Representative images demonstrating luciferase activity in the eye 24 hours post-injection. (B) Luciferase activity plotted for each group over time. Data are presented as mean ± SD. A two-way ANOVA, Tukey’s multiple comparisons test was used for analysis. *p<0.05. n = 3–6. (C) Representative bright field (top) and tdTomato (bottom) fundus images for each group taken 7 days post-injection. (D) Quantification of tdTomato intensity, represented as a fold change compared to PBS. Data are presented as mean ± SD. An ordinary one-way ANOVA, with Tukey’s correction for multiple comparisons test was used for comparisons. *p<0.05. n = 3–6. (E) Representative confocal images of immunohistochemistry showing RFP expression in the Müller glia, the ONH and the TM. n.s.—not significant, LNP- lipid nanoparticle, RFP-red fluorescent protein, ONL- outer nuclear layer, INL-inner nuclear layer, GCL- ganglion cell layer, ONH-optic nerve head, TM-trabecular meshwork, CB-ciliary body.

To determine the impacts of PEGylation and reduced size on LNP penetration from the vitreous to the outer retina, LNPs were delivered intravitreally at a high (Figs 5 and 6) and low dose (S2 and S3 Figs). First, LNP kinetics were measured with a luciferase assay (Figs 5A and 6A, S4 Fig). In general, for all particles, luciferase activity in the eye was measurable at 4 hours post-injection, increased to a maximum level at 24 hours post-injection and decreased by 48 hours post-injection (Figs 5B and 6B). At 24 hours post-injection, 0.5% PEG particles modulated against cholesterol demonstrated significant 2.7-fold, 3.0-fold and 17.5-fold increases in luciferase activity compared to 1.5%, 3% and 5% PEG particles, respectively (Fig 5B, p<0.05). At the same time point, 0.5% PEG particles modulated against DSPC mediated a less robust response, only showing a 1.5-fold and 5.2-fold increase in luciferase activity compared to 3% and 5% PEG particles, respectively (Fig 6B, p<0.05). The 1.5% PEG particles modulated against DSPC showed similar magnitudes of activity to 0.5% PEG particles and mediated a significantly higher level of luciferase activity compared to 5% PEG particles (5.6-fold, Fig 6B, p<0.05). When comparing the luciferase expression between 0.5% PEG particles modulated against cholesterol with 0.5% PEG particles modulated against DSPC, modifications in cholesterol mediated a 2.9-fold and 8.4-fold increase in expression compared to DSPC at 24- and 48-hours post-injection, respectively. For all eight particles, fundus images showed patchy patterns of expression localized around the optic nerve head (ONH) and blood vessels, traditional of inner retinal cell expression (Figs 5C and 6C). When tdTomato intensity was analyzed, PEG particles modulated against cholesterol showed no significant differences between the groups (Fig 5D, p>0.05). In contrast, 0.5% PEG particles modulated against DSPC averaged a 2.4-fold increase in tdTomato intensity compared to PBS controls, which was a significant increase compared to the tdTomato intensity mediated by 1.5% and 5% PEG particles (Fig 6D, p<0.05). Immunohistochemistry, probing for RFP, demonstrated that protein expression was localized to the Müller glia, the ONH and the trabecular meshwork (TM), regardless of modifications (Figs 5E and 6E). When LNPs were delivered at a low dose, RFP expression was localized to the Müller glia and the TM (S2 and S3 Figs).

## Discussion

There is a critical need to develop non-viral gene delivery vehicles for the retina. Even though there are over 20 gene therapy clinical trials utilizing viral vectors for retinal degenerations [4], it is well understood that viral vectors are unable to solely treat all of the genetic forms of retinal degeneration due to cost, packaging capacity and immune response [5]. Additionally, generating penetrating particles that can transfect the outer retina post-intravitreal delivery would generate a paradigm shift moving away from detaching fragile degenerating retinas during subretinal surgery. To optimize the existing FDA approved LNPs for the retina, studies are needed that investigate pharmacokinetics and intracellular mechanisms of LNPs post-ocular delivery. Our studies, first evaluating many cationic and ionizable lipids [13], and now investigating the impacts of size and PEGylation, aim to understand the critical LNP components needed for optimal retina internalization and penetration to the eye. In this paper, we show that particles with less PEG (0.5%) and larger in size (~150 nm) facilitate the highest levels of protein expression post-subretinal and intravitreal delivery. Furthermore, we demonstrate that neither apoE adsorption, nor MERTK dependent phagocytosis are critical for LNP intracellular delivery to the RPE. Finally, we demonstrate that our LNPs can transfect Müller glia, the ONH and the TM post-intravitreal delivery which could have significant impacts for retinal degeneration and glaucoma.

### Intracellular delivery

We utilized a subretinal injection, which places LNPs in close proximity to the RPE to focus on LNP internalization. Our data suggests that particles with 0.5% PEG and ~150 nm in size facilitate the best intracellular delivery in the RPE post-subretinal delivery (Figs 2 and 3). These data are consistent with previous studies that demonstrate 1) LNPs with 0.5 mol % PEG-DMA mediated the highest FVII gene silencing in hepatocytes [25, 30] and 2) that while increasing the PEG content makes particles smaller it also makes them more stable [31, 32]. We hypothesize that the 3% and 5% particles aren’t shedding enough PEG-lipid to allow for sufficient cellular uptake, which is inhibiting the transfection efficiency. In addition, the enhanced efficiency observed with 0.5% PEG particles is most likely due to a combination of particle size and surface composition. Larger particle size allows for more mRNA encapsulation per particle, whereas the mol % of PEG on the particle surface impacts protein adsorption and disassembly for endosomal escape [26, 27]. Interestingly, when comparing DSPC versus cholesterol modifications, increasing DPSC content on the particle surface increased expression by 6-fold. We hypothesize that by decreasing the mol % of PEG and increasing surface DSPC, protein adsorption is enhanced allowing for more efficient internalization [30]. Previous studies established an apoE–LDLR dependent internalization pathway for LNP-mediated expression in the liver after systemic delivery [27]. Successful RPE transfection in *apoE*^*-/-*^ mice after subretinal delivery of LNP-mCherry suggests that apoE, an essential endogenous targeting ligand for the liver, is not required for LNP internalization in the RPE. Since the subretinal injection delivers the LNPs to the RPE, it’s possible that targeting ligands are unnecessary, or alternatively, there are still unknown RPE specific ligands facilitating cellular entry. We also hypothesized that phagocytosis could be playing a role in LNP uptake in the RPE. However, the LNP-mediated expression observed in *Mertk*^*-/-*^ mice, which lack the main receptor required for phagocytosis [29], post-subretinal injection did not support this hypothesis. This finding is consistent with previous studies that demonstrate phagocytosis is dependent on nanoparticle size [23]. Our particles are ~150 nm in size, which is outside the range for phagocytosis (>1000 nm), but within the range for endocytosis (<500 nm). Overall, the exact endocytic process for LNP uptake in the RPE still remains elusive. Different internalization pathways as well as the LNP protein corona and adsorption in the retina needs further investigation.

### Penetration

In order to evaluate LNP penetration, we utilized an intravitreal injection, which releases the LNPs in the vitreous chamber. We hypothesized that smaller particles would allow for penetration from the vitreous chamber to the RPE. However, our data showed that larger particles (~80 nm to 150 nm in size) mediated a higher magnitude of expression than smaller particles (~50 nm to 60 nm in size) and transfection was limited to the inner retina and did not reach the outer retina (more discussion on cellular tropism below) (Figs 5 and 6). As stated above, it’s feasible that increasing the PEG content increases stability and decreases cellular uptake, which is inhibiting the transfection efficiency. Future studies can focus on generating smaller particles through microfluidic processing instead of increasing PEG content to separate these confounding factors of size and stability. Interestingly, when comparing surface modifications, increasing the mol % of cholesterol mediated an ~3-fold (at 24 hours) and ~8-fold (at 48 hours) increase in magnitude of expression compared to DSPC. Although cholesterol has been closely linked to enhancing endosomal escape [33], our data suggest that in the retina having more cholesterol potentially on the surface could enhance penetration, which could be linked to protein adsorption as the particle moves through the retina. It is well understood that LNP size, stability, surface charge and ligands will all work together to generate penetrating LNPs [17]. We now add that surface composition including mol % of cholesterol is an important determinant and that 1.5 mol % of PEG or less is crucial for penetration in the eye.

### Cell tropism

LNP modifications did not alter cellular tropism, but the route of delivery altered cell specific expression. Post-subretinal delivery, all expression was observed in the RPE, which we have previously reported (Figs 2–4) [13]. The OLM, which has an ~30 Angström pore radius (in rabbits), is a junction between the Müller glia and the outer nuclear layer (ONL) which acts as a barrier to the inner retina post-subretinal delivery [16]. It is possible that our smallest particle (~50 nm in size) was still unable to traverse the OLM to allow for transfection in the ONL. When we transitioned to intravitreal delivery, the Müller glia, the ONH and the TM were successfully transfected regardless if particles were 150 nm or 50 nm. Although the ILM prevents penetration into the retina, the Müller glia endfeet sit at the interface of the vitreous humor and the ILM and are susceptible to LNP uptake. Müller glia are a critical therapeutic target for retinal degeneration. With the recent success of reprogramming Müller glia into functional rod photoreceptors, Müller glia remain key targets for regenerating the retina [34, 35]. Although the ONH sits at the back of the eye, the ILM is not part of the ONH composition and it becomes thinner as the retinal interface gets closer to the ONH, which decreases the difficulty of transducing the ONH. Moving to the anterior part of the eye, the TM is located in the angle of the eye and is responsible for regulating the outflow of aqueous humor [36]. The TM is most likely transduced as the particles get circulated out of the vitreous chamber. The TM and the ONH are commonly under investigation to better understand and treat glaucoma [36–38]. Specific to the TM, researchers have been investigating and developing gene therapy approaches that reduce ocular pressure [37]. As glaucoma is a multifaceted disease, researchers have also developed a method of small molecule delivery to the ONH of rats that allows for exploration of molecular and cellular pathways involved in neuropathies and treatment screening [38]. In future studies our LNPs could be used to target the Müller glia, the ONH and the TM to aid in treatment development for retinal degeneration and glaucoma.

Our long-term goal is to generate penetrating LNPs that can transfect the outer retina post-intravitreal delivery. Coincidently, by generating better penetrating LNPs, we will be able to enhance transfection efficiency after other important ocular delivery methods for retinal degeneration including subretinal and suprachoroidal delivery [39]. Decreasing particle size by increasing PEG content failed to improve LNP penetration. Instead, the use of 0.5% or 1.5% PEG is optimal for retinal transfection. In the future, we will generate smaller particles without the addition of PEG. Furthermore, solely adjusting the LNP composition (PEG, cholesterol, DSPC, ionizable lipid) may not be enough to alter the LNP transfection profile. The discovery and addition of RPE and photoreceptor targeted ligands may be required to generate LNPs that can penetrate through the vitreous. Our future studies will focus on the generate of these novel ligands and understanding the critical LNP characteristics required for effective retinal transfection.

## Supporting information

S1 Fig Luciferase activity post-subretinal injection. Representative images showing luciferase activity in the eye at 4 hours (A & C) and 48 hours (B & D) post-injection.
(TIF)

S2 Fig Low dose intravitreal PEG & Chol. (A) Representative bright field (top) and tdTomato (bottom) fundus images for each group taken 7 days post-injection. (B) Quantification of tdTomato intensity, represented as a fold change compared to PBS. Data are presented as mean ± SD. An ordinary one-way ANOVA, with Tukey’s correction for multiple comparisons test was used for comparisons. n = 3–6. (C) Representative confocal images of immunohistochemistry showing RFP expression in the Müller glia and the TM. n.s.—not significant, LNP- lipid nanoparticle, RFP-red fluorescent protein, ONL- outer nuclear layer, INL-inner nuclear layer, GCL- ganglion cell layer, TM-trabecular meshwork, CB-ciliary body.
(TIF)

S3 Fig Low dose intravitreal PEG & DSPC. (A) Representative bright field (top) and tdTomato (bottom) fundus images for each group taken 7 days post-injection. (B) Quantification of tdTomato intensity, represented as a fold change compared to PBS. Data are presented as mean ± SD. An ordinary one-way ANOVA, with Tukey’s correction for multiple comparisons test was used for comparisons. n = 3–6. (C) Representative confocal images of immunohistochemistry showing RFP expression in the Müller glia and the TM. n.s.—not significant, LNP- lipid nanoparticle, RFP-red fluorescent protein, ONL- outer nuclear layer, INL-inner nuclear layer, GCL- ganglion cell layer, TM-trabecular meshwork, CB-ciliary body.
(TIF)

S4 Fig Luciferase activity post-intravitreal injection. Representative images showing luciferase activity in the eye at 4 hours (A & C) and 48 hours (B & D) post-injection.
(TIF)
