# Supplementary figures and images for: The effects of PEGylation on LNP based mRNA delivery to the eye
Source: PLoS One. 2020 Oct 29;15(10):e0241006. doi: 10.1371/journal.pone.0241006 (PMC7595320; doi:10.1371/journal.pone.0241006)

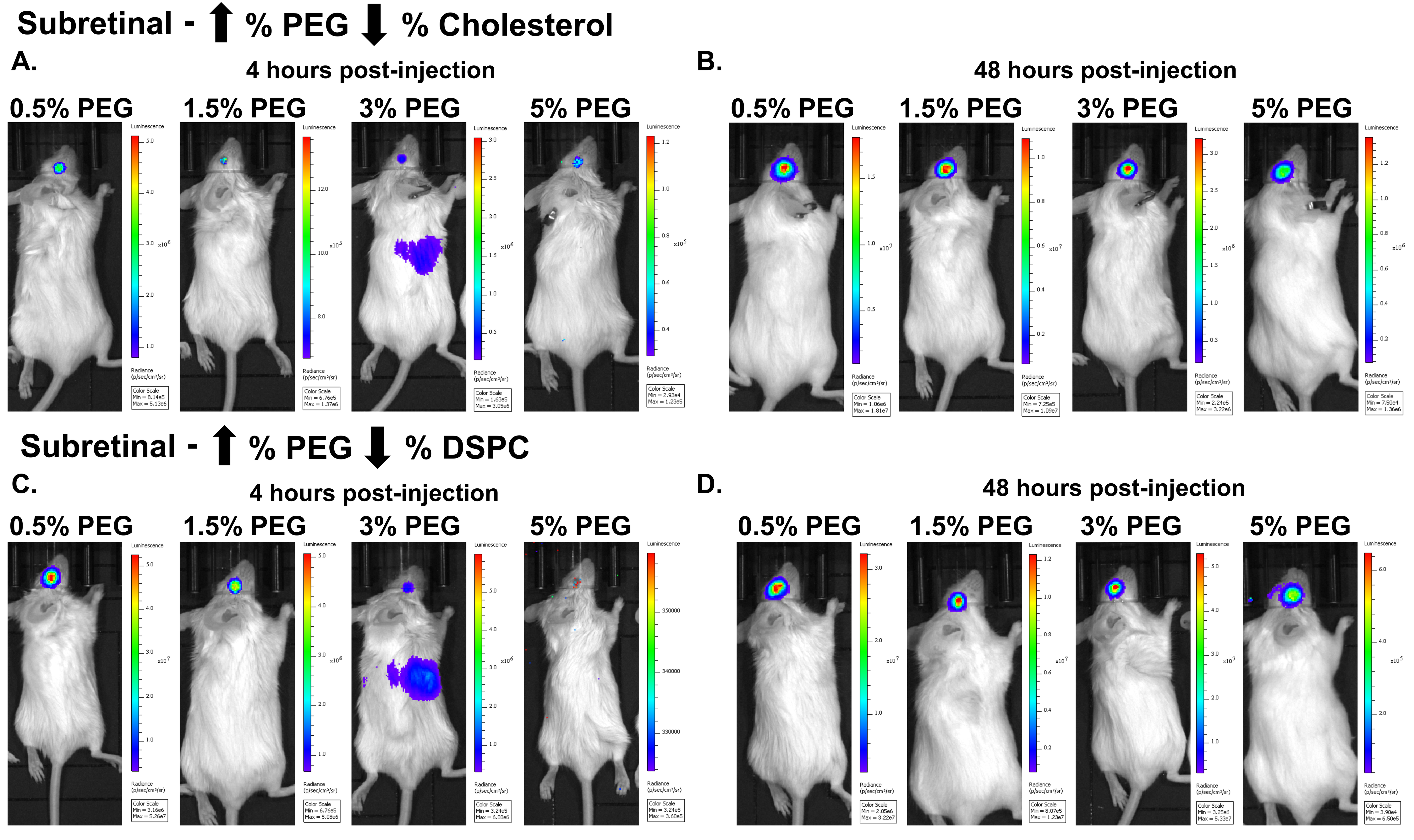

Supplement: S1 Fig — Representative images showing luciferase activity in the eye at 4 hours (A & C) and 48 hours (B & D) post-injection. (TIF) [file pone.0241006.s001.tif]

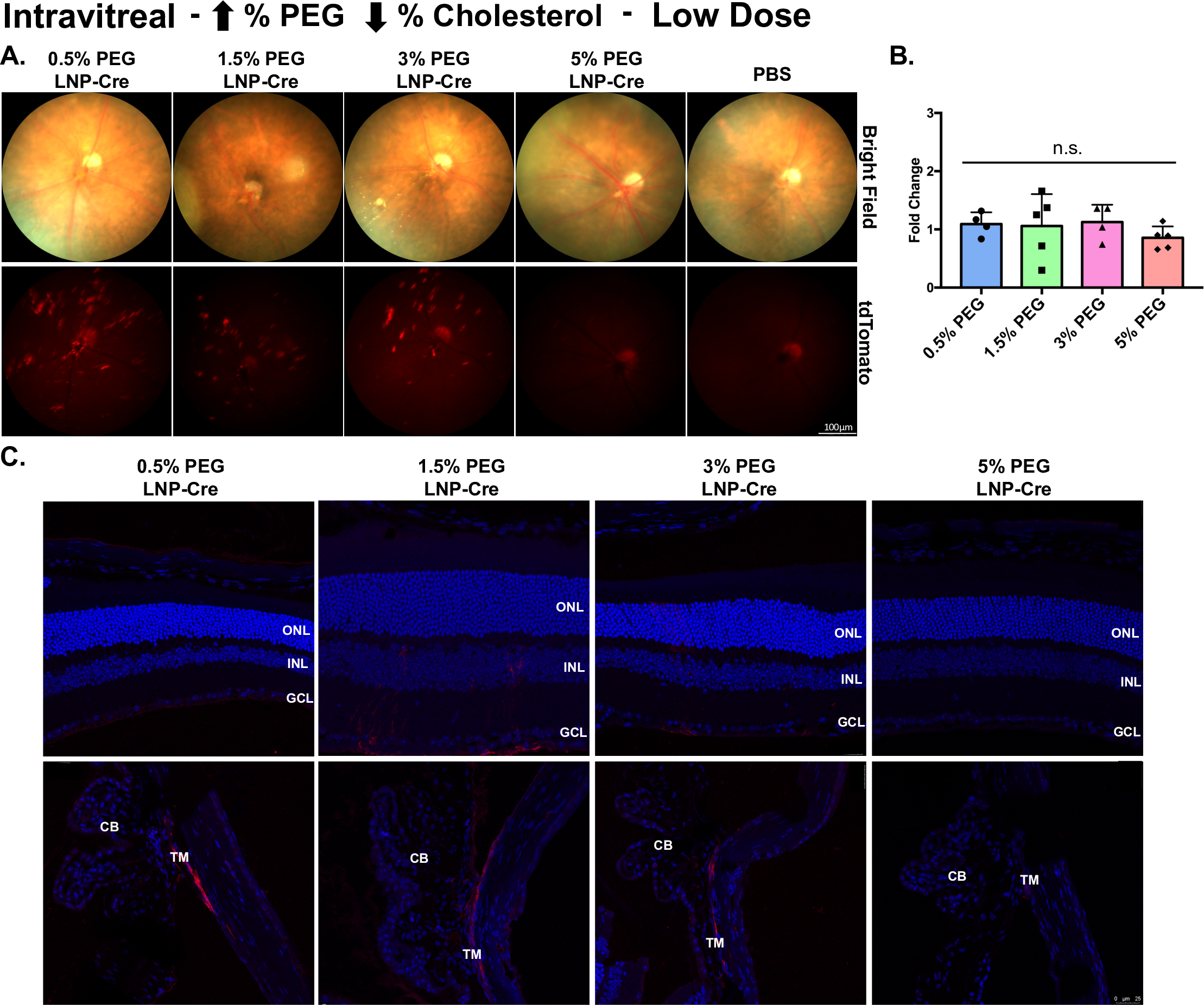

Supplement: S2 Fig — (A) Representative bright field (top) and tdTomato (bottom) fundus images for each group taken 7 days post-injection. (B) Quantification of tdTomato intensity, represented as a fold change compared to PBS. Data are presented as mean ± SD. An ordinary one-way ANOVA, with Tukey’s correction for multiple comparisons test was used for comparisons. n = 3–6. (C) Representative confocal images of immunohistochemistry showing RFP expression in the Müller glia and the TM. n.s.—not significant, LNP- lipid nanoparticle, RFP-red fluorescent protein, ONL- outer nuclear layer, INL-inner nuclear layer, GCL- ganglion cell layer, TM-trabecular meshwork, CB-ciliary body. (TIF) [file pone.0241006.s002.tif]

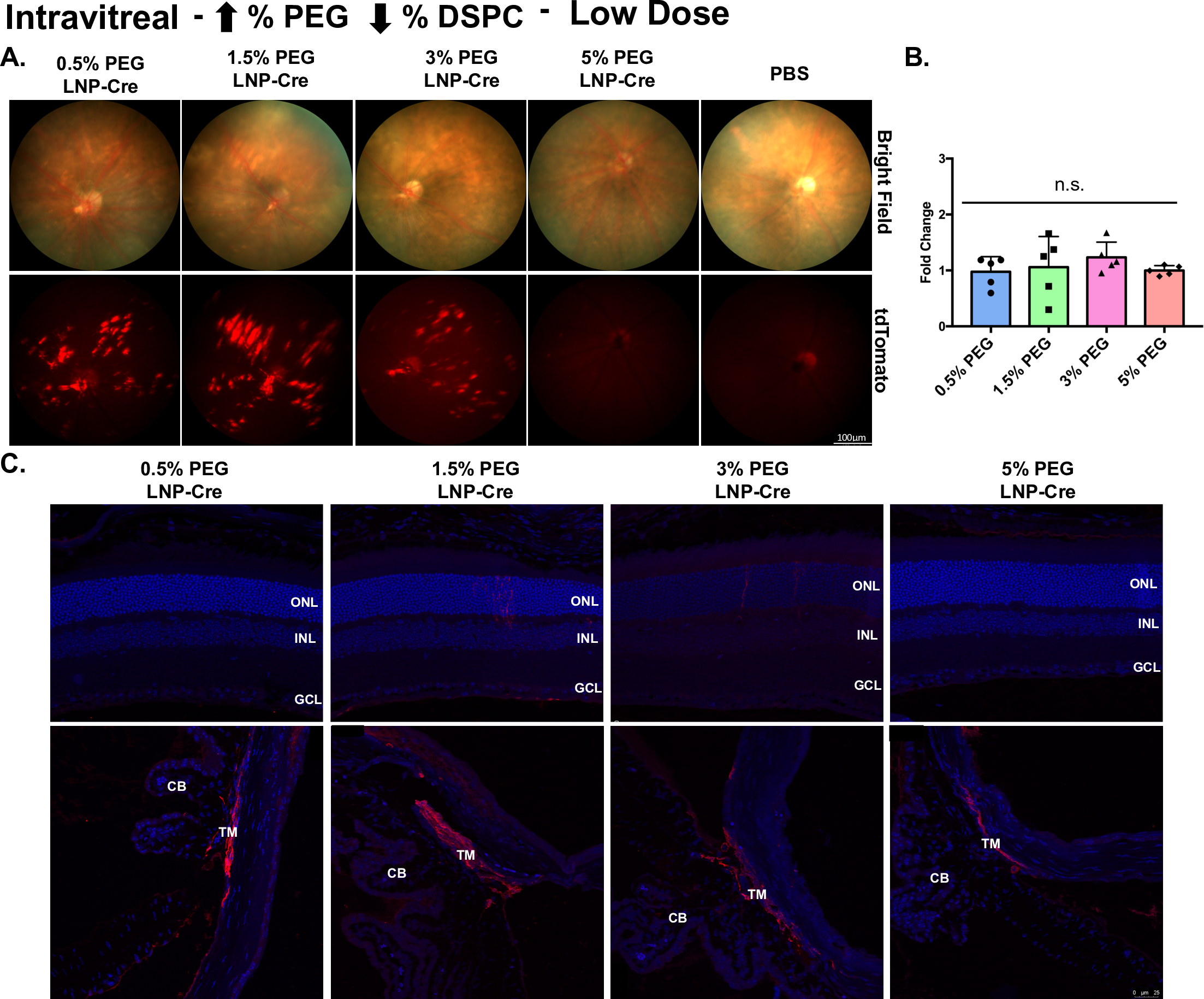

Supplement: S3 Fig — (A) Representative bright field (top) and tdTomato (bottom) fundus images for each group taken 7 days post-injection. (B) Quantification of tdTomato intensity, represented as a fold change compared to PBS. Data are presented as mean ± SD. An ordinary one-way ANOVA, with Tukey’s correction for multiple comparisons test was used for comparisons. n = 3–6. (C) Representative confocal images of immunohistochemistry showing RFP expression in the Müller glia and the TM. n.s.—not significant, LNP- lipid nanoparticle, RFP-red fluorescent protein, ONL- outer nuclear layer, INL-inner nuclear layer, GCL- ganglion cell layer, TM-trabecular meshwork, CB-ciliary body. (TIF) [file pone.0241006.s003.tif]

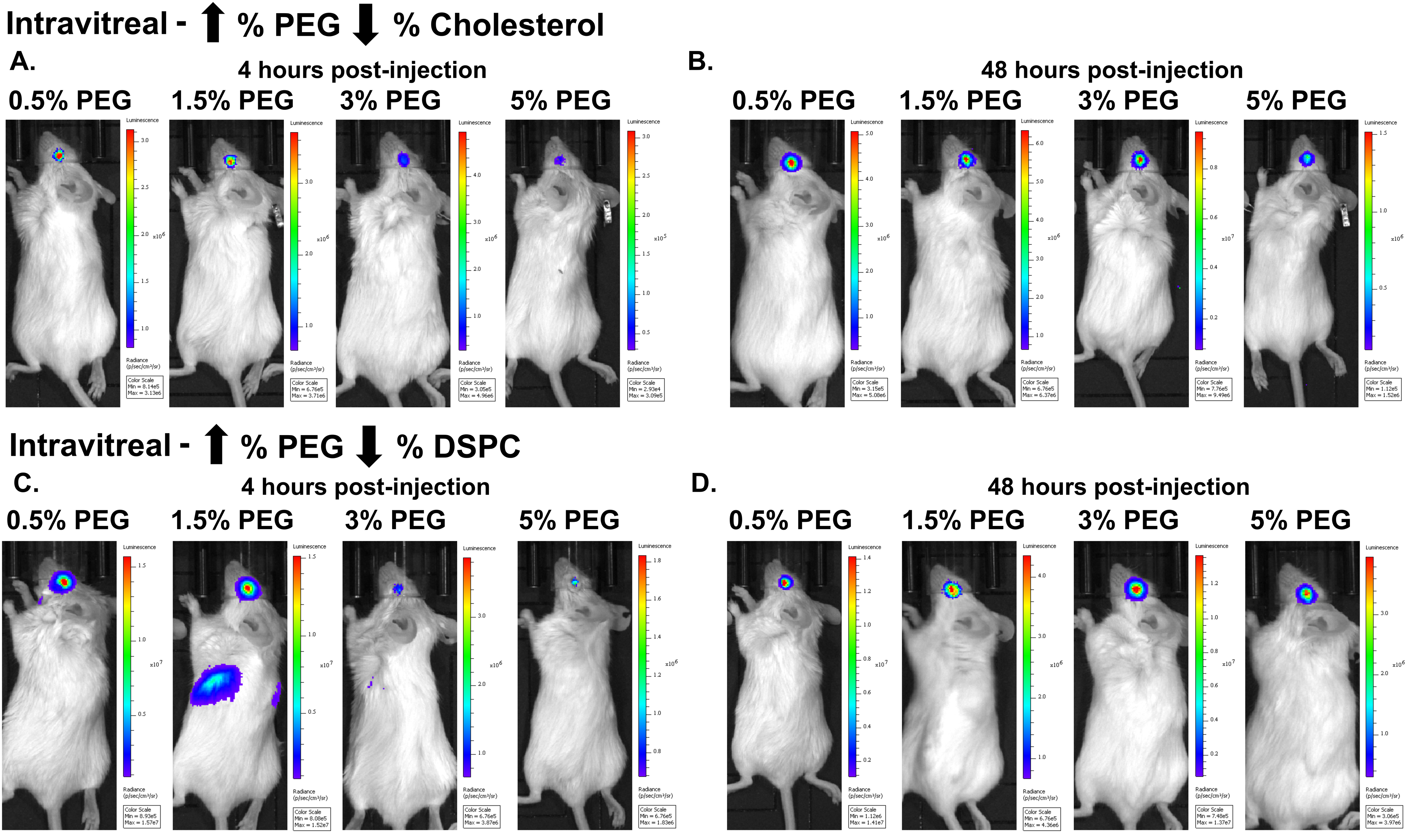

Supplement: S4 Fig — Representative images showing luciferase activity in the eye at 4 hours (A & C) and 48 hours (B & D) post-injection. (TIF) [file pone.0241006.s004.tif]
